# Hsp90 Inhibitors Exhibit Resistance-Free Antiviral Activity against Respiratory Syncytial Virus

**DOI:** 10.1371/journal.pone.0056762

**Published:** 2013-02-27

**Authors:** Ron Geller, Raul Andino, Judith Frydman

**Affiliations:** 1 Department of Biology, Stanford University, Stanford, California, United States of America; 2 Department of Microbiology and Immunology, University of California San Francisco San Francisco, California, United States of America; Centro de Biología Molecular Severo Ochoa (CSIC-UAM), Spain

## Abstract

Respiratory syncytial virus (RSV) is a major cause of respiratory illness in young children, leading to significant morbidity and mortality worldwide. Despite its medical importance, no vaccine or effective therapeutic interventions are currently available. Therefore, there is a pressing need to identify novel antiviral drugs to combat RSV infections. Hsp90, a cellular protein-folding factor, has been shown to play an important role in the replication of numerous viruses. We here demonstrate that RSV requires Hsp90 for replication. Mechanistic studies reveal that inhibition of Hsp90 during RSV infection leads to the degradation of a viral protein similar in size to the RSV L protein, the viral RNA-dependent RNA polymerase, implicating it as an Hsp90 client protein. Accordingly, Hsp90 inhibitors exhibit antiviral activity against laboratory and clinical isolates of RSV in both immortalized as well as primary differentiated airway epithelial cells. Interestingly, we find a high barrier to the emergence of drug resistance to Hsp90 inhibitors, as extensive growth of RSV under conditions of Hsp90 inhibition did not yield mutants with reduced sensitivity to these drugs. Our results suggest that Hsp90 inhibitors may present attractive antiviral therapeutics for treatment of RSV infections and highlight the potential of chaperone inhibitors as antivirals exhibiting high barriers to development of drug resistance.

## Introduction

Respiratory syncytial virus (RSV) is the leading cause of acute lower respiratory infections. In children under the age of 5, it is estimated that RSV results in 3.4 million severe infections requiring hospitalization worldwide and 66,000–199,000 deaths [1]. RSV is also recognized as an important pathogen in the elderly, where it leads to 170,000 infections and 10,000 deaths in the US alone [2]. No RSV vaccine is currently available; furthermore, the development of such a vaccine presents significant challenges due to the difficulties associated with inducing immune responses in infants and the elderly [3], [4]. Similarly, no effective antivirals are available to combat RSV infections [5], [6]. Prophylactic treatment with monoclonal antibodies has been shown to be effective against RSV, although their use remains cost prohibitive and limited to high-risk infants [5], [6]. Therefore, the identification of novel antivirals for treatment of RSV infections remains a top priority.

RSV belongs to the paramyxovirus family, which includes many important human pathogens such as human parainfluenza (HPIV), mumps, and measles viruses [7], [8]. All paramyxoviruses are enveloped and have a linear, single-stranded, negative-sense RNA genome [7], [8]. The genome of RSV is ∼15 kb and encodes 11 proteins [7], [8]. In virions, the viral genome is bound by the nucleocapsid (N) protein and 3 proteins that are required for initiation of viral replication upon entry into the cell: the P phosphoprotein, the M2-1 transcription processivity factor, and the large polymerase subunit L [7], [8]. The 250 kDa L protein encodes the RNA-dependent RNA polymerase, a multi-domain protein required for genome replication, viral mRNA synthesis, as well as mRNA capping and polyadenylation [7], [8]. Following infection of epithelial cells in vitro, RSV mRNAs and proteins can be detected within 4–6 hours [7], [8]. Virus release is observed at 10–12 hours post infection, peaks at 24 hours, and continues until cell death 30–48 hours post infection. Infection with RSV results in numerous alterations in cellular gene expression, including changes in the levels of transcripts encoding cytokines and chemokines, as well as several cellular protein folding factors, such as Hsp70 and Hsp90 [9]–[11].

Hsp90 is a highly conserved and essential molecular chaperone at the center of a large protein-folding network [12]–[14]. Together with a cohort of cochaperones, Hsp90 regulates the maturation and activity of a large set of client proteins, including many signaling and regulatory proteins such as kinases, hormone receptors, and tumor suppressor proteins. The importance of these client proteins to regulation of cellular activity has made Hsp90 an attractive target for anticancer therapy and several specific Hsp90 inhibitors are currently undergoing clinical evaluation for cancer treatment [13], [15], [16]. Pharmacological inhibition of Hsp90 blocks the maturation of its client proteins, thereby targeting them for degradation by the ubiquitin-proteasome pathway [12], [13]. Hsp90 is also used by numerous DNA and RNA viruses to mediate the activity and maturation of various viral proteins (reviewed in [17], [18]). Accordingly, Hsp90 inhibitors display broad-spectrum antiviral activity. Most antiviral drugs eventually elicit drug-resistant viral variants that escape inhibition, which is one of the major hurdles to effective antiviral therapy [19]–[21]. Intriguingly, drug-resistance did not emerge when Hsp90 inhibitors were used to block poliovirus replication, suggesting that these types of inhibitors may be refractory to the development of drug resistance [19]–[21]. The broad-spectrum antiviral activity of Hsp90 inhibitors and their low propensity for eliciting drug resistance make Hsp90 inhibitors attractive candidates for antiviral therapy.

Hsp90 inhibitors have been shown to reduce the replication of several negative-sense RNA viruses, including the paramyxovirus HPIV [22]. In this work, the viral RNA-dependent RNA polymerases of these viruses, the L proteins, were shown to be degraded upon Hsp90 inhibition, suggesting a common mechanism for the antiviral activity of Hsp90 inhibitors in negative-sense RNA viruses. However, whether the L protein of RSV is degraded upon Hsp90 inhibition remains to be demonstrated. More recently, Hsp90 was found to localize to sites of RSV replication and virion assembly within the cell [23]. Moreover, treatment of RSV infected cells with an Hsp90 inhibitor was shown to reduce viral spread, indicating Hsp90 inhibitors possess antiviral activity against RSV. No Hsp90-dependent RSV protein was identified, however, and the effects on virus production were not quantitated.

The lack of antivirals to combat RSV infection prompted us to evaluate the potential of Hsp90 inhibitors as antiviral therapeutics against RSV replication. In this study, we set out to characterize the antiviral activity of Hsp90 inhibitors against both laboratory and clinical isolates of RSV in standard tissue culture cells as well as in an *in vivo* relevant model of primary differentiated human airway epithelial cells (HAEC). We find that two clinically relevant Hsp90 inhibitors, 17-allylamino-17-demethoxygeldanamycin (17AAG) and 17-dimethylaminoethylamino-17-demethoxygeldanamycin (17DMAG), possess antiviral activity against both laboratory and clinical isolates of RSV and that Hsp90 inhibitors display potent antiviral activity against RSV in the in vivo relevant HAEC model. Immunoprecipitation of viral proteins using a polyclonal RSV antibody raised against the viral particle revealed that Hsp90 inhibition reduces the abundance of a single viral protein; this protein corresponded in size to that of the viral polymerase, the L protein, implicating it as an Hsp90 client protein. Finally, we find that extensive growth of RSV under conditions of Hsp90 inhibition does not select for viral mutants resistant to 17AAG, indicating that Hsp90 inhibitors may be generally refractory to the development of drug resistance. These results suggest that the RSV L protein is an Hsp90 client and highlight the potential of Hsp90 inhibitors as antivirals against RSV infections.

## Results

**Figure 1:**
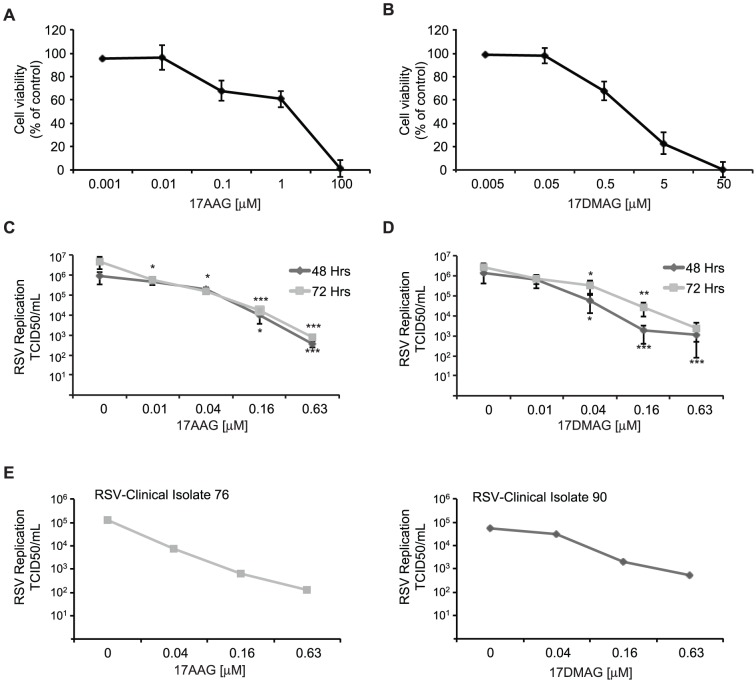
Hsp90 inhibitors have anti-RSV activity at non-toxic concentrations. (A–B) Toxicity of 17AAG (A) or 17DMAG (B) in HEp-2 cells was assessed after 72 hours using the Alamar blue cytotoxicity assay. (C–D) Hsp90 inhibitors reduce RSV replication. HEp-2 cells infected with RSV-A2 at an MOI of 0.01 were treated with the indicated concentrations of 17AAG (C) or 17DMAG (D) for 72 hours prior to determination of virus production. Data represent the mean 50% tissue culture infective dose per mL (TCID50/mL) and SEM of ≥3 experiments. (E) Antiviral activity of Hsp90 inhibitors against clinical isolates of RSV in HEp-2 cells. Cells infected with clinical isolates 76 and 90 were treated with the indicated concentrations of the Hsp90 inhibitors 17AAG and 17DMAG, respectively. * p<0.05, ** p<0.01, *** p<0.001.

Hsp90 inhibitors have been shown to reduce the replication of numerous viruses in tissue culture (reviewed in [17], [18]). To examine whether RSV is dependent on Hsp90 activity, we tested the effect of 2 clinically relevant Hsp90 inhibitors, 17AAG and 17DMAG, on the replication of RSV in HEp-2 cells. We first established the sensitivity of uninfected HEp-2 cells to these Hsp90 inhibitors to obtain a range of drug concentrations that is appropriate for testing antiviral activity (Figure 1A, B). HEp-2 cells were grown in the absence or presence of increasing concentrations of Hsp90 inhibitors and toxicity was evaluated after 72 hours using the Alamar blue assay, which quantitatively assesses cell proliferation by measuring cellular metabolic activity. Hsp90 inhibitors did not significantly reduce cell viability at concentrations up to 0.01 µM and 0.05 µM for 17AAG and 17DMAG, respectively (p>0.65; Figure 1A, B). To investigate whether these Hsp90 inhibitors displayed antiviral activity against RSV, HEp-2 cells were infected with the A2 laboratory strain of RSV at a multiplicity of infection (MOI) of 0.01 and incubated in the presence of increasing concentrations of Hsp90 inhibitors for either 48 or 72 hours. Both Hsp90 inhibitors significantly reduced RSV virus production 48 and 72 hours post infection (Figure 1C, D). At concentrations not affecting cellular viability, 17AAG reduced RSV replication by 50% at 48 and 72 hours post-infection, while 17DMAG treatment resulted >85% reduction in viral production at both time points. Hsp90 inhibitors also had antiviral activity against two clinical isolates of RSV, highlighting the potential of Hsp90 inhibitors for treatment of RSV infections (Figure 1E). Altogether, these results demonstrate that Hsp90 inhibitors possess antiviral activity against both laboratory and clinical isolates of RSV at concentrations that are not toxic to host cells.

**Figure 2:**
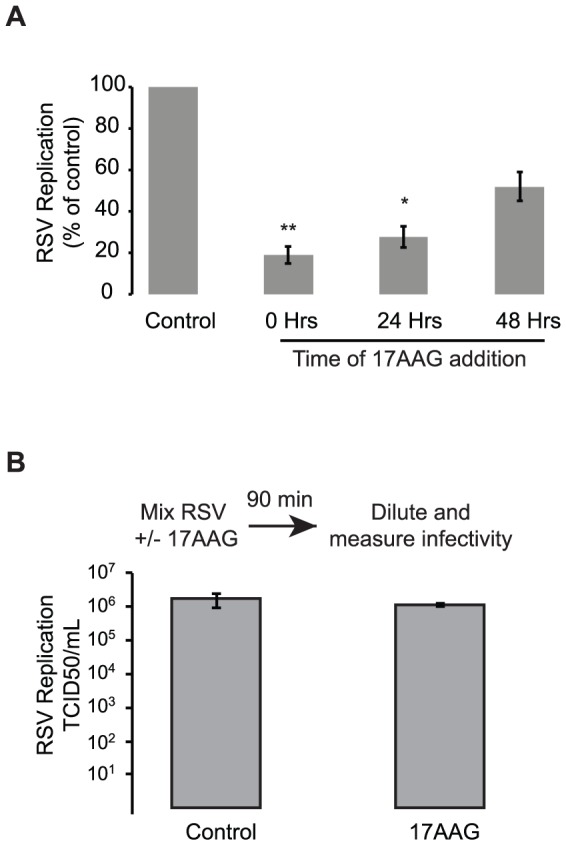
Mechanism of action underlying the antiviral activity of Hsp90 inhibitors against RSV. (A) Hsp90 inhibitors do not act via inhibition of early steps in the RSV cycle, and significantly reduce RSV replication even when added 24 hours post infection. HEp-2 cells were infected with RSV-A2 at an MOI of 1. 17AAG (0.1 µM) was added at 0, 24, or 48 hours post infection and virus production measured 72 hours post infection. Data represents mean and SEM of 4 experiments. (B) The antiviral activity of Hsp90 inhibitors is independent of direct effects on virus infectivity. RSV was incubated with 1 µM 17AAG or DMSO as a control for 90 minutes at 4°C prior to infectivity determination using endpoint titration. Data represent the mean TCID50/mL and SEM of 3 experiments.

We next investigated the mechanism underlying the antiviral activity of Hsp90 inhibitors. RSV can infect cells either by extracellular infection or, following initial infection events, also by direct cell-to-cell spread, leading to the formation of syncytia. To test whether Hsp90 inhibitors retain their antiviral activity following initial infection events, we investigated the effect of 17AAG treatment on virus production at different times post infection (Figure 2A). Cells were infected with RSV and 17AAG was added at 0, 24, or 48 hours post infection. An MOI of 1 was used in order to achieve more homogenous infection of the cells and avoid multiple cycles of infection during the course of the experiment. The effect of drug addition on virus production was assessed 72 hours post infection. 17AAG significantly reduced viral replication even when added 24 hours following infection, suggesting Hsp90 inhibitors interfere with virus replication and spread at stages subsequent to initial infection events (Figure 2A). To ensure that the Hsp90 inhibitors were acting via modulation of Hsp90 activity and not by physically reducing RSV infectivity, RSV was incubated with 17AAG or DMSO as a control for 90 minutes prior to determination of virus infectivity (Figure 2B). Pretreatment of RSV with 17AAG did not reduce virus infectivity, indicating the antiviral activity of Hsp90 inhibitors was independent of any direct virucidal activity (Figure 2B).

**Figure 3:**
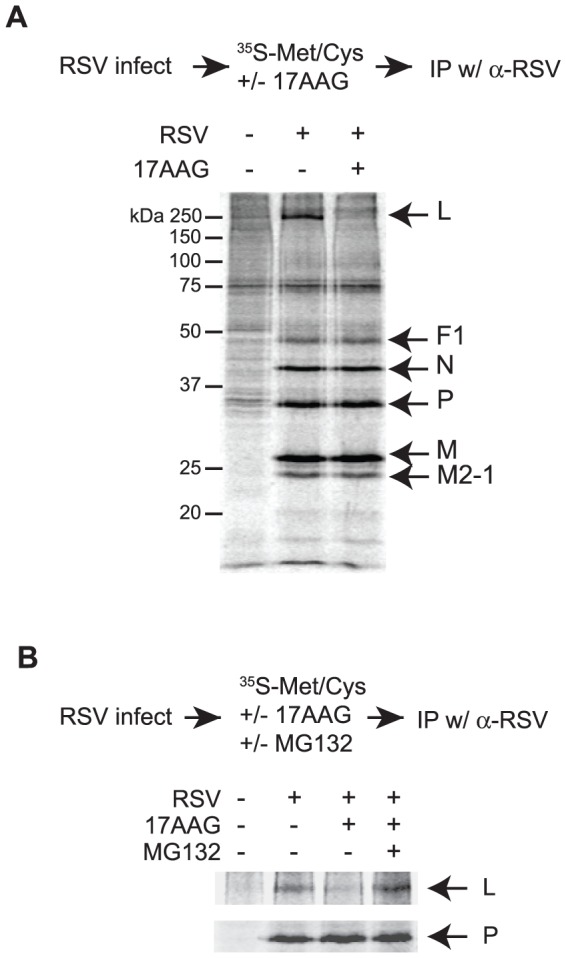
The RNA polymerase of RSV is a likely Hsp90 client protein. (A) Hsp90 inhibitors destabilize a viral protein of 250 kDa, corresponding to the size of the RSV polymerase, the L protein. Control or RSV-infected HEp-2 cells were treated with 17AAG or DMSO and labeled with ^35^S-methionine/cysteine. Viral proteins were immunoprecipitated, separated by 12% SDS-PAGE, and visualized by autoradiography. The Hsp90 inhibitor 17AAG specifically reduced the intensity of the 250 kDa protein by ∼60%. (B) Hsp90 inhibition leads to proteasomal degradation of the viral L protein. Control or RSV-infected HEp-2 cells were treated with 17AAG, 17AAG and the proteasome inhibitor MG132, or DMSO and labeled with ^35^S-methionine/cysteine. The reduction of L protein caused by 17AAG is abrogated by MG132. Viral proteins were immunoprecipitated, separated by 12% SDS-PAGE, and visualized by autoradiography. Densitometry analysis showed a band intensity of 40% and 75% for 17AAG treatment and the combined 17AAG/MG132 treatment, respectively, relative to control conditions. ** p<0.01, *** p<0.001.

Many Hsp90 client proteins become destabilized upon inhibition of Hsp90 and thus targeted for degradation by the proteasome [12], [13], [24]. This effect has proven useful for identifying Hsp90-clients among viral proteins [19], [22]. We exploited this feature to identify which RSV proteins are clients of Hsp90 (Figure 3). To examine whether any viral proteins become destabilized upon inhibition of Hsp90, control or RSV-infected cells were radiolabeled in the presence or absence of 17AAG. Viral proteins were then immunoprecipitated using a polyclonal antibody raised against the viral particle and their abundance examined by autoradiography. Uninfected cells served as a control for non-specific binding of cellular proteins. Several viral proteins were specifically immunoprecipitated from infected cells but not control cells and their identity deduced according to their expected molecular weight (Figure 3A) [7]. Inhibition of Hsp90 resulted in the specific degradation of a single ∼250 kDa protein of viral origin, which was not observed in uninfected cells. This band corresponds in molecular weight to the RSV L protein, the viral RNA-dependent RNA polymerase (Figure 3A), implicating it as an Hsp90 client protein. A hallmark of Hsp90 clients is that they are targeted for proteasomal degradation upon treatment with Hsp90 inhibitors [12], [13]. Accordingly, we tested whether treatment with the proteasome inhibitor MG132 impaired the reduction in the levels of the 250 kDa viral protein observed upon 17AAG treatment. This 17AAG-induced protein degradation was blocked by addition of the proteasome inhibitor MG132, indicating that, as observed for other Hsp90 client proteins, the lack of Hsp90 activity leads to proteasomal degradation of this viral protein (Figure 3B) [12], [13]. These results suggest that the RSV polymerase is an Hsp90 client protein and are in agreement with previous findings where Hsp90 inhibition has been shown to result in the degradation of viral polymerases from a broad range of viruses, including other negative-strand RNA viruses [17], [18], [22].

**Figure 4:**
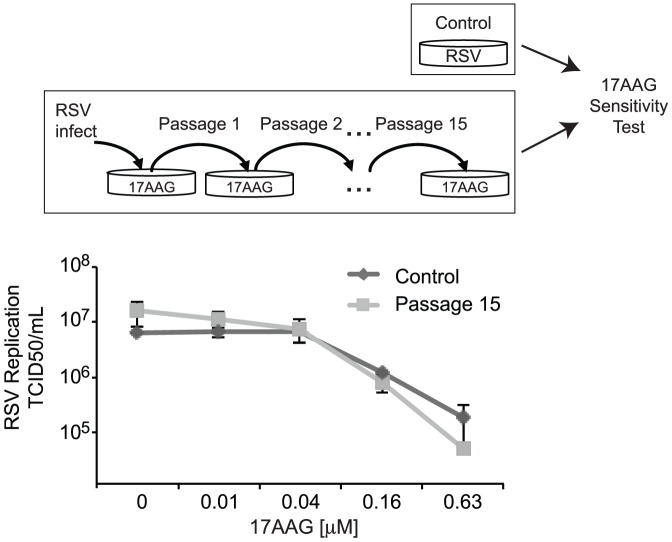
Extensive growth of RSV in the presence of Hsp90 inhibitors does not select for the emergence of drug resistance. Antiviral activity of 17AAG against an RSV viral population obtained following 15 passages in the presence of 17AAG and a control RSV viral population. HEp-2 cells were infected with the 17AAG passaged viral population or a control viral population and treated with the indicated concentration of 17AAG for 72 hours. Virus production was then assayed by endpoint dilution. Data indicate the mean TCID50/mL and SEM of ≥2 experiments.

Acquisition of drug resistance by RNA viruses can rapidly render antiviral compounds ineffective and remains a major challenge in antiviral therapy [20], [21], [25]. This is enabled by the high mutation rates of RNA viruses, which allow them to rapidly adapt to different selective pressures, including antiviral therapies [25]–[27]. We have previously reported that the RNA virus poliovirus was unable to develop drug resistance to Hsp90 inhibitors [19]. As the mechanism of action of Hsp90 inhibitors against poliovirus involves effects on the viral capsid and not the polymerase, as in the case for RSV, we investigated if extensive growth of RSV in the presence of Hsp90 inhibitors will select for RSV mutants which are resistant to Hsp90 inhibition. RSV-A2 was serially passaged in the presence of 17AAG for 15 passages. We used a dose escalation regimen to select for drug resistance to the Hsp90 inhibitor, starting with a low drug concentration inhibiting ∼50% of virus production (0.0078 µM 17AAG) and doubling the drug concentration with each passage until a dose resulting in near maximal viral inhibition was reached (0.25 µM 17AAG). Following passage 15, the sensitivity of the resulting viral population to 17AAG was compared to that of a control virus not grown in the presence of an Hsp90 inhibitor. Notably, the passage 15 virus was equally as sensitive to Hsp90 inhibitors as the control virus (Figure 4), indicating that prolonged treatment with Hsp90 inhibitors does not select for drug resistant viral mutants. These results further generalize previous findings that Hsp90 inhibitors are refractory to the development of drug resistance and emphasize the potential of such antiviral approach for treatment of RNA virus infections.

**Figure 5:**
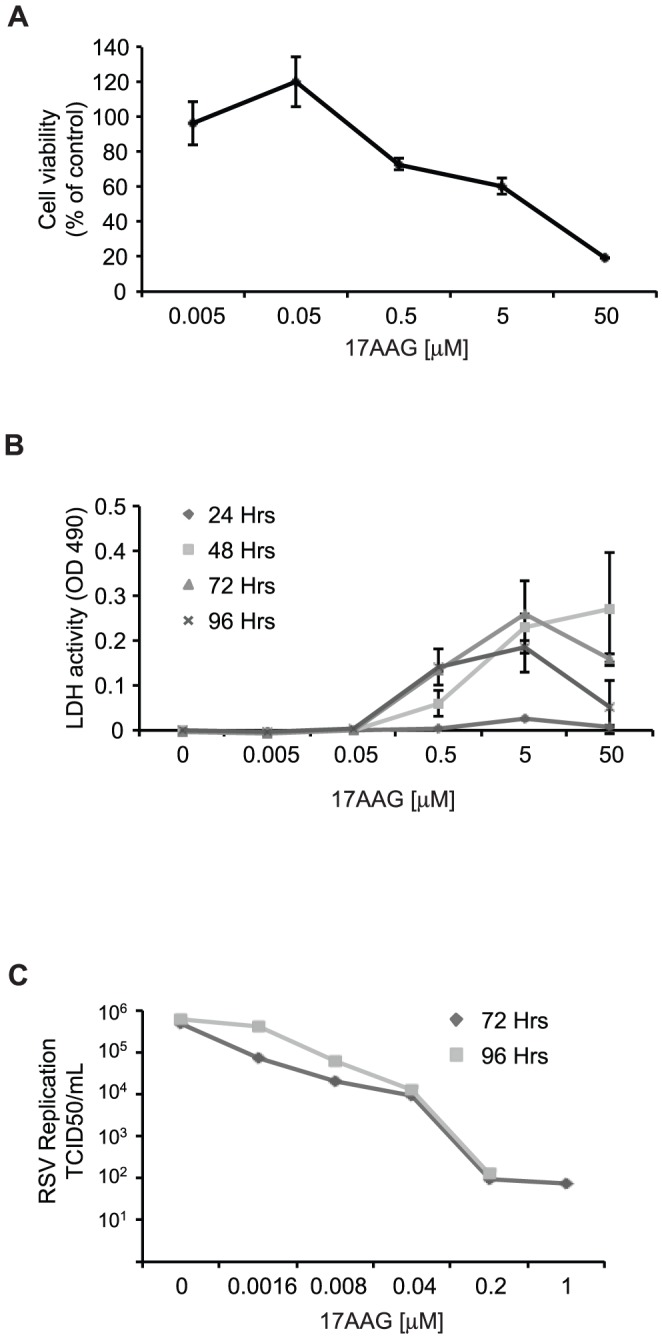
Antiviral activity of Hsp90 inhibitors against RSV in well-differentiated primary human airway epithelial cells. (A–B) Cytotoxicity of 17AAG in well-differentiated human airway epithelial cells (HAEC). HEAC were treated with the indicated concentrations of 17AAG and toxicity measured after 96 hours using the Alamar blue assay (A) or at 24, 48, 72, and 96 hours using the lactate dehydrogenase (LDH) cytotoxicity assay (B). (C) Antiviral activity of Hsp90 inhibitors in HEAC. HAEC were infected with RSV-A2 and treated with the indicated concentrations of 17AAG for 72 hours. Virus production was then assayed by endpoint dilution.

In humans, RSV primarily infects epithelial cells of the airways and the nasopharynx [7]. Differentiated primary human airway epithelial cells (HAEC) grown at an air-liquid interface constitute a highly relevant system for studying RSV replication and are consequently a good model for assessing the pharmacological potential of Hsp90 inhibitors against RSV [28]. We therefore tested the effect of 17AAG on RSV replication in HAEC. We first evaluated the sensitivity of HAEC to Hsp90 inhibition to identify a range of drug concentrations that is appropriate for evaluating antiviral activity (Figure 5A, B). HAEC that were grown at an air-liquid interface for over 2 weeks were treated with increasing concentrations of 17AAG for 96 hours, and toxicity was evaluated using the Alamar blue assay. Previous studies have shown that 17AAG maintains its effect on Hsp90 during these incubation periods [29]. No toxicity was observed at concentrations below 0.05 µM (Figure 5A and Figure S1A). These results were confirmed using an alternative cytotoxicity assay based on lactate dehydrogenase (LDH) activity, which measures the release of the cytosolic LDH enzyme from damaged cells into the media (Figure 5B). In agreement with results from the Alamar Blue assay, no cytotoxicity was observed at concentrations below 0.05 µM. We next examined the antiviral activity of 17AAG in HAEC. HAEC that were grown at an air-liquid interface for over 2 weeks were infected with RSV and incubated in the absence or presence of increasing concentrations of 17AAG for 72 or 96 hours prior to determination of virus production. 17AAG reduced RSV replication at concentrations as low as 0.0016 µM and at concentrations of 0.04 µM virus production was reduced over 90% at both 72 and 96 hours post infection (Figure 5C). Similar results were obtained using a separate HAEC culture (Figure S1B). These results indicate that Hsp90 inhibitors can potently reduce RSV replication in a highly relevant cell culture model at concentrations not affecting cell viability and highlight the potential of Hsp90 inhibitors for treatment of RSV infections *in vivo*.

## Discussion

Herein, we establish the potential of two clinically relevant Hsp90 inhibitors as antivirals against both laboratory and clinical strains of RSV. Importantly, we show that 17AAG, which has shown promise in advanced clinical evaluation against cancer, reduced RSV replication in an *in vivo*-relevant model of well-differentiated primary human airway epithelial cells at drug concentrations well below those affecting cell viability [13], [15], [16]. In agreement with the reported anti-cancer activity of Hsp90 inhibitors, 17AAG exhibited much lower toxicity in the primary HEAC cells than in immortalized HEp-2 cells (5 fold lower; Figure 1 and Figure 5). Interestingly, despite the reduced sensitivity of primary lung cells to the inhibitor, 17AAG inhibits RSV replication more potently in these cells than in immortalized cells typically used in culture. As a consequence, in primary cells the antiviral activity of 17AAG is more pronounced with reduced toxicity. This finding resonates with our previous observations that Hsp90 inhibitors block poliovirus replication more potently and with lower toxicity in a primary cell model of infection, when compared to immortalized cells [19]–[21]. Together, these observations highlight the feasibility of antiviral therapy using Hsp90 inhibitors in humans. The identification of a non-toxic Hsp90 inhibitor suitable for clinical use may present a general treatment for respiratory viral infections since Hsp90 inhibitors reduce the replication of several of the most important respiratory viral pathogens, namely RSV, HPIV, influenza virus, and rhinovirus [17], [19], [22], [23], [30]. The restriction of these respiratory viruses to the airways should enable direct delivery of Hsp90 inhibitors to the site of infection via inhalation, thereby achieving higher local inhibitor concentrations in the lungs while minimizing toxicity by reducing exposure of non-target organs to the inhibitor.

Many Hsp90 clients are degraded by the proteasome upon Hsp90 inhibition, a finding that has facilitated the identification of new client proteins [12], [13]. Employing a similar strategy, we investigated whether any viral proteins were degraded in infected cells upon treatment with an Hsp90 inhibitor. Indeed, using an antibody specific for the viral particle, immunoprecipitation of lysates from infected cells revealed the degradation of a single protein of approximately 250 KDa upon Hsp90 inhibition (Figure 3). The size of this protein, which directly correlates to that of the viral polymerase, the L protein, and the fact that it was only expressed in infected cells, and thus of viral origin, strongly implicate it as the RSV L protein (Figure 3). Moreover, the dependence of viral polymerases of numerous viruses, including both DNA and RNA viruses, and in particular those of viruses related to RSV, lend support to the notion that the viral polymerase is an Hsp90 client protein (for a review see [17]). However, the lack of commercially available antibodies specific for the RSV L protein precluded us from directly demonstrating the identity of this protein. Further mechanistic and biochemical studies will be required to unambiguously identify the Hsp90-dependent protein and define in detail the role of Hsp90 in the RSV life cycle.

The extreme capacity of RNA viruses for adaption to selective pressures, stemming from error prone replication mechanisms, supports their rapid acquisition of drug resistance to antiviral drugs, and makes antiviral drug resistance one of the principle hurdles to the development of effective antivirals against infections with these viruses [20], [21], [25]–[27]. We have previously demonstrated that poliovirus, an RNA virus shown to gain resistance to numerous antivirals, did not become resistant to Hsp90 inhibitors despite extensive growth in the presence of such inhibitors [19]. Here we similarly find that RSV does not become resistant to Hsp90 inhibitors despite extensive growth in the presence of 17AAG (15 passages; Figure 4). This is in contrast to previously described RSV inhibitors, which yield resistant virus variants within a few passages [31]. The lack of observed drug resistance to Hsp90 inhibitors by these unrelated RNA viruses, each having a different viral function dependent on Hsp90 activity, supports the generality of the finding that Hsp90 inhibitors possess a low propensity for eliciting drug resistance and further highlights the potential of these inhibitors for antiviral therapy.

To achieve its role in maintaining cellular proteostasis, Hsp90 functions in concert with a large cohort of cochaperones that help regulate its activity and client specificity [12], [14], [32]. Targeting these Hsp90 cochaperones for antiviral therapy has the potential to reduce toxicity associated with general Hsp90 inhibition, only blocking specific cellular protein folding pathways required for viral replication. For example, human butyrate-induced transcript 1 was identified as a membrane bound Hsp90 cofactor involved in HCV replication [33], [34]. As the replication of many viruses, and in particular plus-strand RNA viruses, is dependent upon membranes, the inhibition of such membrane-bound Hsp90 cofactors can provide more specific targets for antiviral therapy with reduced toxicity [35]. Future work should aim to identify additional Hsp90 cofactors involved in viral replication and assess their potential for antiviral therapy.

## Materials and Methods

### Cells, viruses, and reagents

HEp-2 cells (CCL-23) were purchased from ATCC and maintained in DMEM/F12 (Invitrogen), 10% FCS, and antibiotics at 37°C. RSV strain A2 (VR-1540) was obtained from ATCC and 2 clinical isolates were a kind gift from Dr. Bruce Patterson (Stanford University). Primary human airway epithelial cells were obtained from either Lonza (SAEC, CC-0294), and grown according to supplier's recommendation, or were obtained as a kind gift of Dr. Walter Finkbeiner (UCSF) after the use of human tissues for this study was approved by The Committee on Human Research at the University of California San Francisco and written informed consent was obtained for sample collection and culture preparation. The cells were grown to confluency in 12-well plates on transwell inserts (Corning, Cat. 3493), then differentiated at an air-liquid interface for 2 weeks prior to use. The hsp90 inhibitors 17-allylamino-17-demethoxygeldanamycin (17AAG) and 17-dimethylaminoethylamino-17-demethoxygeldanamycin (17DMAG) were purchased from LC laboratories and dissolved in DMSO. All Hsp90 inhibitor experiments were conducted under dim lighting conditions with DMSO serving as a control.

### Virus infections

RSV stocks were generated by infecting HEp-2 cells. Upon observation of cytopathic effect (CPE), cells and media were centrifuged to remove cell debris and supernatants containing the virus were frozen in liquid nitrogen. Clinical isolates were passaged twice in HEp-2 cells prior to use. Infections of HEp-2 cell were carried out at 37°C in DMEM/F12 with 2% FCS for 2 hours. Following infection, virus inoculum was removed and the media was replace with media containing the indicated amount of 17AAG or DMSO serving as control. Virus production was determined by endpoint dilution on HEp-2 cells in 96 well plates. Infected cells were identified by visual inspection of CPE. For infection of primary cells, cells were washed with BEBM media (Lonza), and then RSV was applied to the apical side of the cells for 1 hour. Following infection, the viral inoculum was removed and cells washed again. To assess viral output, 0.2 mL of BEBM media was added to the apical side of the cell layer for 10 minutes to collect the virus, and then frozen at −80°C until titration on HEp-2 cells. For assessment of virucidal effects, RSV was incubated with 1 µM 17AAG for 90 minutes at 4°C, prior to endpoint titration on HEp-2 cells.

### Viral passaging

HEp-2 cells were infected with RSV-A2 as described above. Following infection, the media was replaced with media containing 0.0078 µM 17AAG, a concentration inhibiting ∼50% of virus production, and infection allowed to continue until CPE was reached. The resulting virus was then used to inoculate fresh HEp-2 cells and the 17AAG concentration was doubled. This procedure was repeated until a drug concentration of 0.25 µM 17AAG (passage 6), at which point drug concentration was kept constant until 15 passages were completed. The sensitivity of the resulting passage 15 virus to increasing concentrations of 17AAG was then assessed as described above and compared to that of control virus.

### Toxicity evaluation

Fresh media containing 10% Alamar blue (Serotec) was added to cells for 2.5 hours prior to measuring by fluorescence on an M1000 (Tecan) plate reader. LDH cytotoxicity assay (Cayman Chemical) was performed according to manufacturer's protocol with absorbance read at 490 nm.

### Analysis of RSV Proteins

HEp-2 cells in confluent 35 mm plates were infected with RSV at an MOI of ∼5. The cells were then washed and incubated in DMEM/F12 with 2% FCS. After 16 hours, the cells were treated with actinomycin D (1 µg/mL) for 2 hours (Figure 3A). Since actinomycin D treatment inhibits host DNA dependent transcription, but should not affect RSV, this treatment increased labeling of RSV proteins relative to cellular proteins. We obtained similar results in the absence of the drug, albeit with lower virus-specific band intensity. The media was then replaced with pulse media (cysteine and methionine free DMEM with 5% dialyzed FCS) containing actinomycin D and either 17AAG (5 µM), 17AAG (5 µM) or DMSO as a control for 30 minutes prior to addition of ^35^S-methionine/cysteine (100 µCi/mL) for 2 hours. Cells were then washed and lysed in RIPA buffer containing protease inhibitors. Viral proteins were immunoprecipitated using 5 µg of goat polyclonal anti-RSV antibody raised against the RSV particle (Fitzgerald Industries International catalog number 20-RG45), separated on a 12% SDS-PAGE gel, and visualized by autoradiography. Precision plus protein prestained standards (Bio-rad Laboratories) were run on the gel to estimate protein sizes. For analysis of protein degradation, a similar protocol was used but MG132 (20 µM) was included to block the proteasome, and no actinomycin D was used. Densitometry analysis was conducted using ImageJ software version 1.47b.

### Statistical analysis

For determining significance of antiviral effects, virus titers were log transformed prior to performing a two-tail t test using Microsoft Excel software.

## Supporting Information

Figure S1 **Antiviral activity and toxicity of Hsp90 inhibitors in primary human airway epithelial cells.** (A) Cytotoxicity of 17AAG in HAEC. HEAC grown at an air-liquid interface for 2 weeks were treated with the indicated concentrations of 17AAG and toxicity measured 72 hours post infection using the Alamar blue assay. (B) Antiviral activity of Hsp90 inhibitors in HEAC. HAEC were infected with RSV-A2 and treated with the indicated concentrations of 17AAG for 72 hours prior to determination of virus production by endpoint dilution.
(EPS)
